# Publisher Correction: Partial proteasomal degradation of Lola triggers the male-to-female switch of a dimorphic courtship circuit

**DOI:** 10.1038/s41467-020-14881-1

**Published:** 2020-02-20

**Authors:** Kosei Sato, Hiroki Ito, Atsushi Yokoyama, Gakuta Toba, Daisuke Yamamoto

**Affiliations:** 1 Neuro-Network Evolution Project, Advanced ICT Research Institute, National Institute of Information and Communications Technology (NICT), 588-2 Iwaoka, Nishi-ku, Kobe 651-2492 Japan; 2 Tohoku University Graduate School of Life Sciences, Sendai, 980-8577 Japan; 3 Tohoku University Graduate School of Medicine, Sendai, 980-8575 Japan; 4 Research Administration/Management Office, University of Tsukuba, Tsukuba, 305-8577 Japan

**Keywords:** Developmental biology, Genetics, Neuroscience

Correction to: *Nature Communications* 10.1038/s41467-018-08146-1, published online 11 January 2019.

The original version of this article incorrectly omitted the received/accepted dates of Received: 4 June 2018; Accepted: 11 December 2018. This has now been corrected in the PDF and HTML versions of the article.

