# Supplementary material for: Partial proteasomal degradation of Lola triggers the male-to-female switch of a dimorphic courtship circuit
Source: Nat Commun. 2019 Jan 11;10:166. doi: 10.1038/s41467-018-08146-1 (PMC6329818; doi:10.1038/s41467-018-08146-1)
Supplement: Supplementary file 1 — Supplementary Information [file 41467_2018_8146_MOESM1_ESM.pdf]

Supplementary Information

Partial proteasomal degradation of Lola triggers the male-to-female switch of a dimorphic courtship circuit

Sato et al.

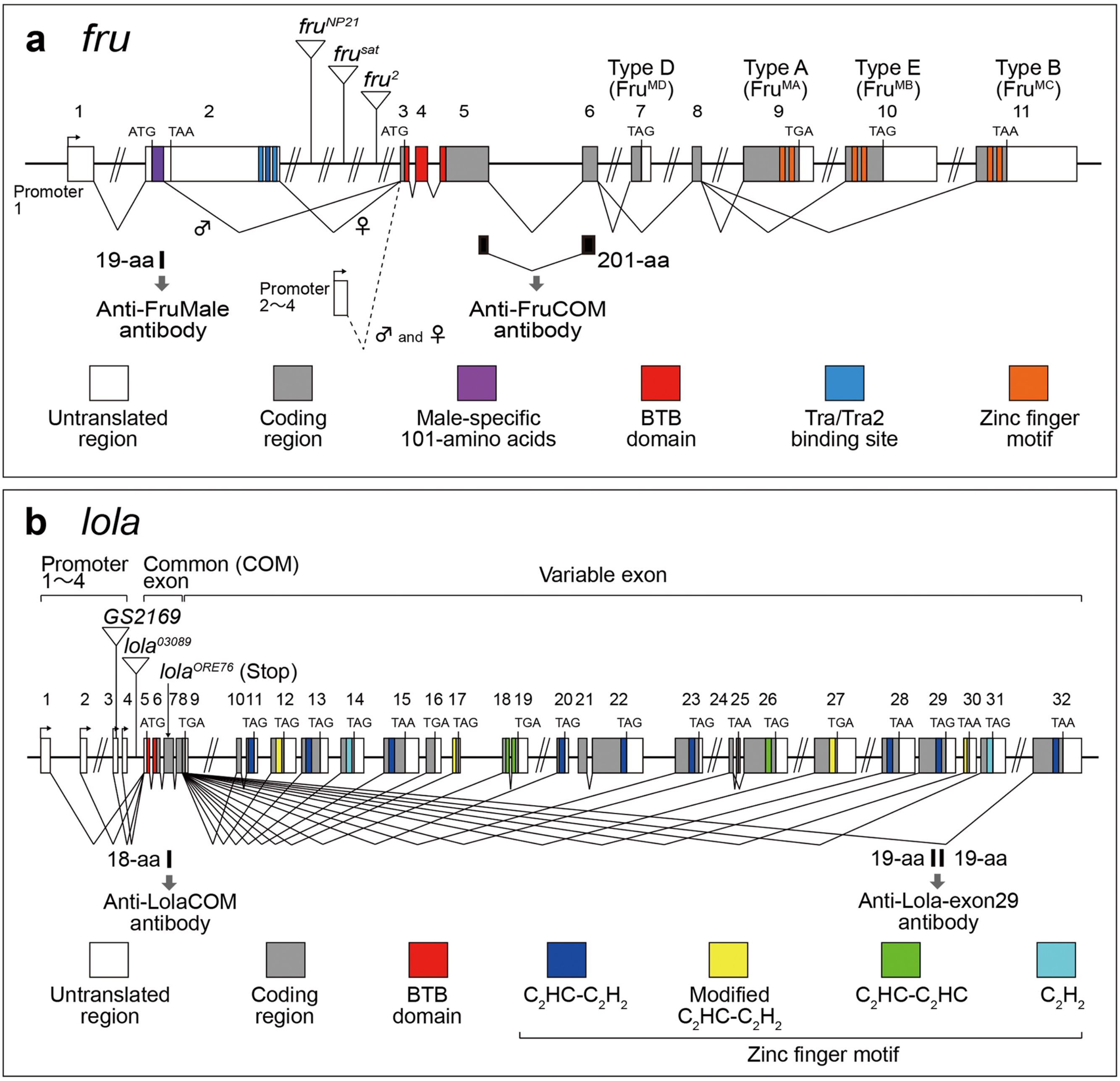

Supplementary Figure 1: The *fru* and *lola* loci.

a, The *fru* locus and exon (box)-intron (thin line) organization of isoform types A, E, and B with different zinc-finger motifs and isoform type D without a zinc-finger motif. The types A, E and B in our study correspond to isoforms A, B and C of other research groups, respectively. The P-insertions (triangles), promoters (P1-P4) and exons (1-11) are indicated. The regions containing epitopes for the anti-Fru antibodies are indicated. **b**, Schematic representation of the genomic organization of the *lola* locus. The entire locus consists of 32 exons. Transcription starts at either of the 5' variable exons (exons 1-4), and 3' variable exons (exons 9-32) are alternatively spliced to the constant exons (exons 5-8), generating variants encoding 20 Lola isoforms with distinct C-terminal domains containing zinc finger motifs. The protein coding regions recognized by the anti-Lola antibodies are indicated. The exon-intron organization and possible splicing patterns are drawn based on Goeke et al.[1] and Ohsako et al.[2] for *lola* and Billeter et al.[3] and Ito et al.[4] for *fru*.

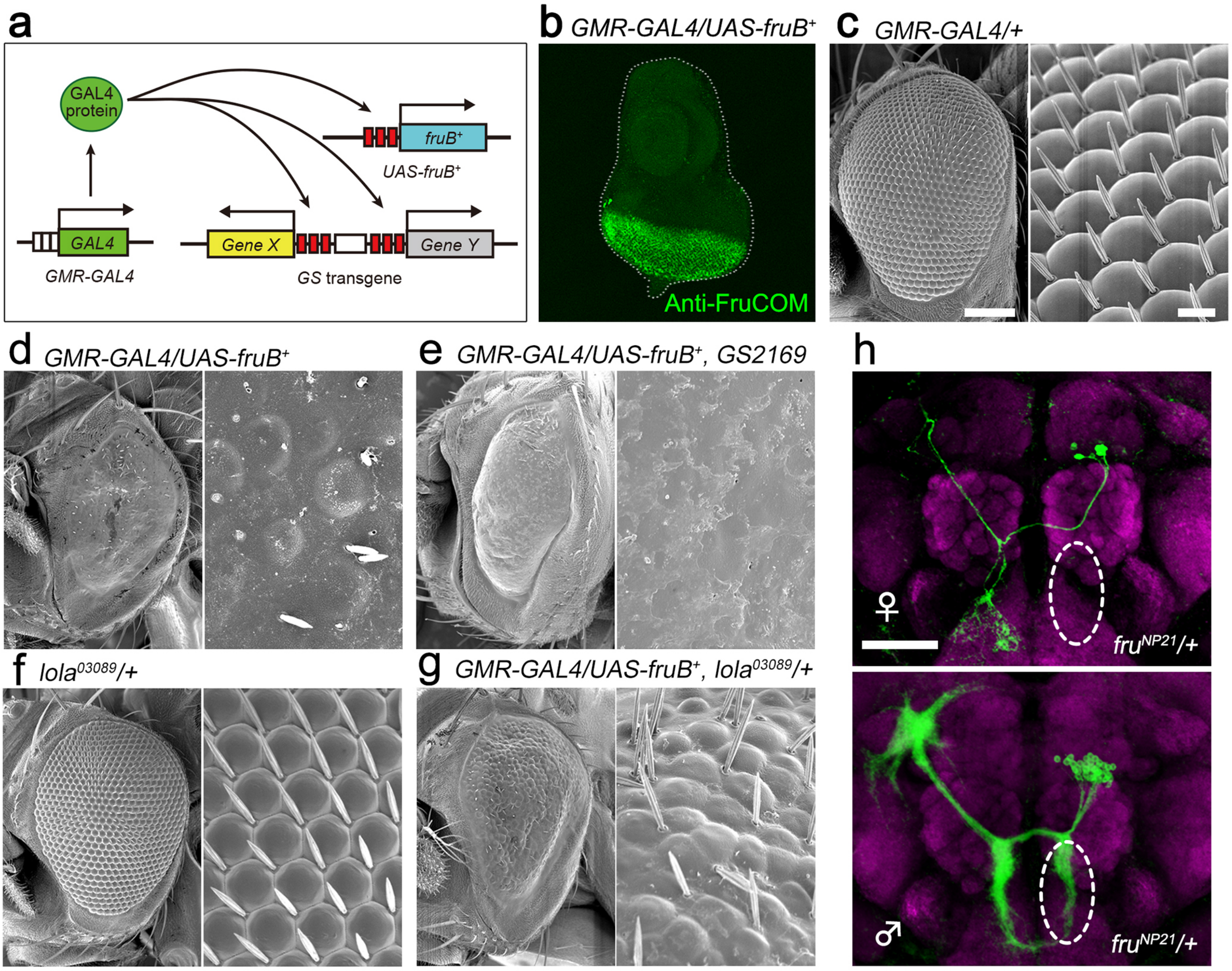

Supplementary Figure 2: Phenotypes associated with heterologous and endogenous Fru expression. **a-g**, The eye structure defects induced by *fru+*-overexpression and its modification by *lola* gene dosage. **a**, Strategy to recover *fru* modifiers using the GeneSearch (GS) system. **b**, An eye-antennal disc with ectopic FruB expression. **c-g**, The compound eye of a control fly carrying only *GMR-GAL4* (**c**), flies in which *GMR-GAL4* was used to overexpress *fru+* type B alone (**d**) or together with *GS2169* (**e**), that of *lola* heterozygotes without (**f**) or with *fru+*-type B overexpression as driven by *GMR-GAL4* (**g**) The scale bar shown in panel **c** applies to panels **c-g**. **h**, Neuroblast clones of the sexually dimorphic mAL neurons in the female (upper panel) and male (lower panel) brain. The ipsilateral neurite is present only in the male (circled with a dotted line). Scale bars: 100 μm (left-hand side) and 10 μm (right-hand side) in (**c-g**); 50 μm in (**h**).

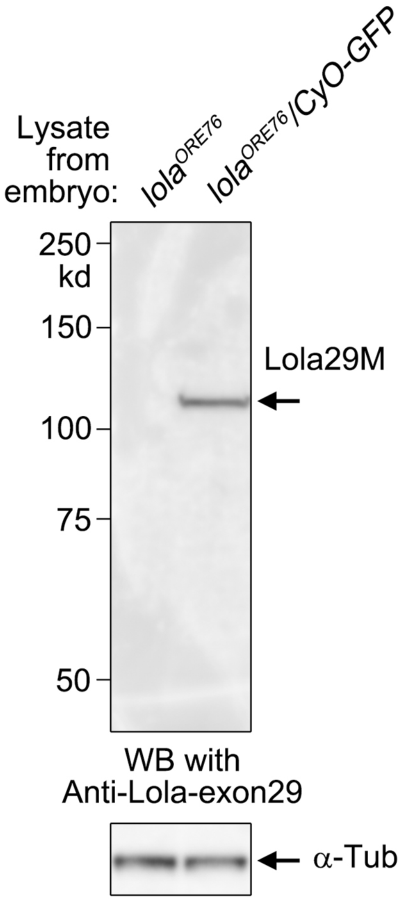

Supplementary Figure 3: Lola expression detected by an anti-Lola-Exon 29 antibody.

Western blot analysis of lysates prepared from *lola* homozygous (left-hand side lane) and heterozygous (right-hand side lane) embryos as probed by the anti-Lola-exon 29 antibody, demonstrating that this antibody specifically recognizes Lola29M. α-Tubulin served as a loading control. Source data are provided as a Source Data file.

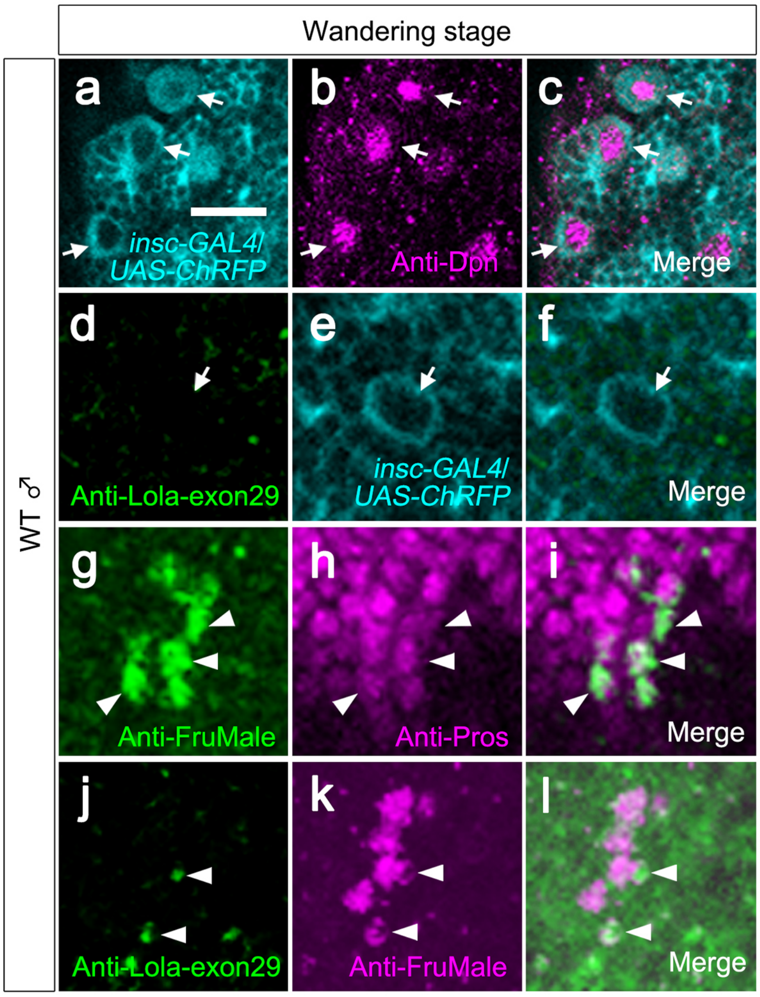

Supplementary Figure 4: Lola29M is coexpressed with FruM in the wandering-stage larval CNS.
a-c, CNS cells in a wandering-stage larva labeled by the combination of *UAS-ChRFP* and *insc-GAL4* (**a**) are immunopositive for the anti-Deadpan (Dpn) antibody (**b**), indicating that *insc-GAL4* serves as a neuroblast marker. A merged image is shown in (**c**). **d-l**, Immunostaining of CNS cells from wild-type larvae at the wandering stages with anti-FruMale, anti-Prospero (Pros), and anti-Lola-exon 29 antibodies. Pros is a marker for ganglion mother cells (GMCs) differentiating into neurons and not expressed in neuroblasts. Neuroblasts are indicated with arrows and differentiating GMCs and neurons are shown with arrowheads. FruM was detectable in some GMCs and neurons (**g-i**) but not in neuroblasts. The anti-Lola-exon 29 antibody that specifically recognizes Lola29M/F labeled some of the differentiating GMCs and neurons (**j-l**) but none of the neuroblasts (**d-f**). Scale bar: 10 μm.

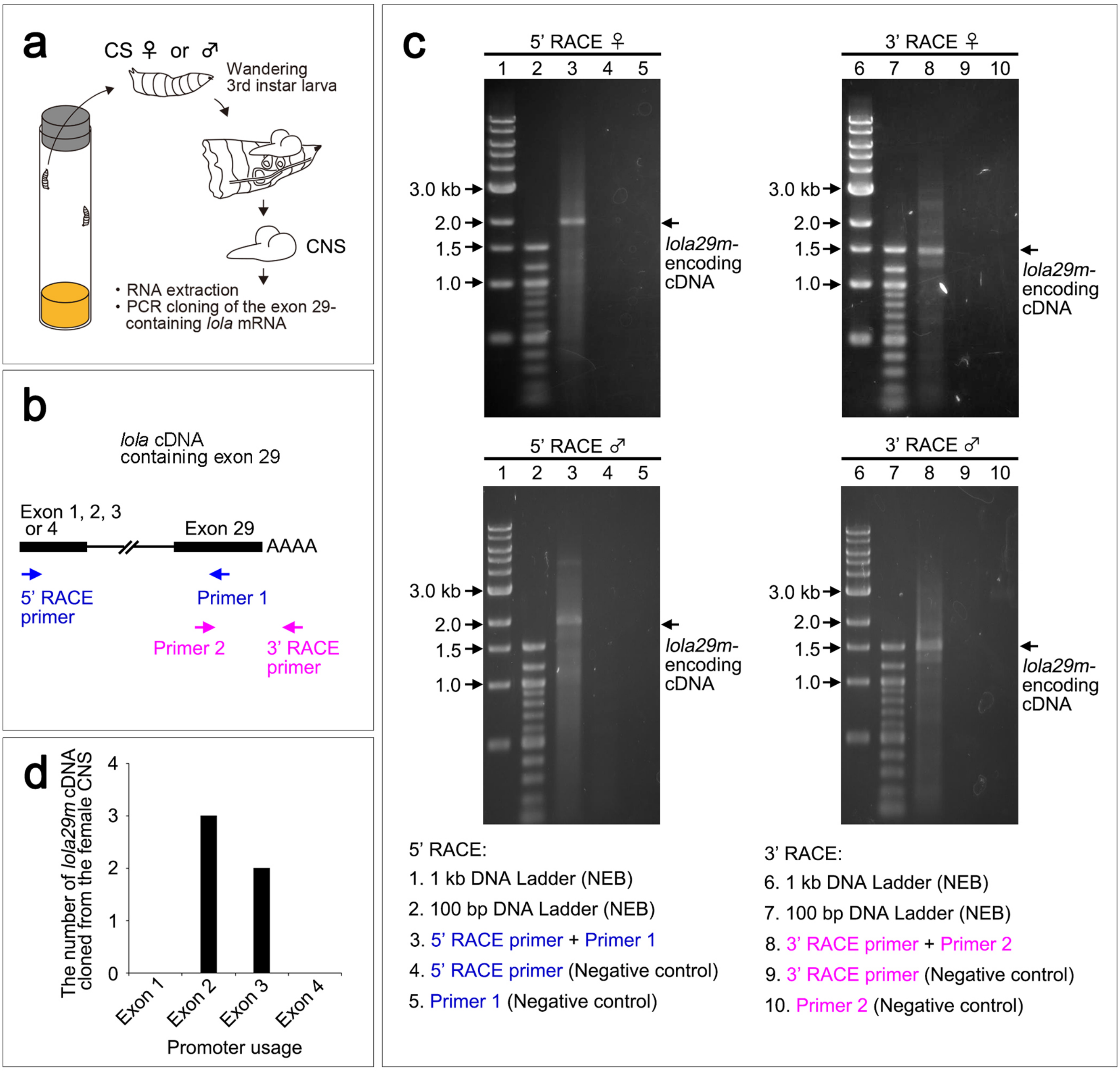

Supplementary Figure 5: No sex difference in promoter usage for transcripts containing the sequence from exon 29.

a, Flow diagram for the steps of 5' and 3' RACE experiments. **b**, Primer design for 5' and 3' RACE experiments to obtain full length cDNAs for exon 29-containing transcripts. **c**, 5' and 3' RACE PCRs with female-derived RNAs each yielded cDNAs that encoded sequences identical to the male transcripts. **d**, Full-length cDNAs contained either exon 2 or exon 3 at the 5'-most segment, indicating that the exon 29-containing primary RNAs are transcribed by two different promoters immediately upstream of exon 2 and exon 3, respectively, in both female and males. Source data are provided as a Source Data file.

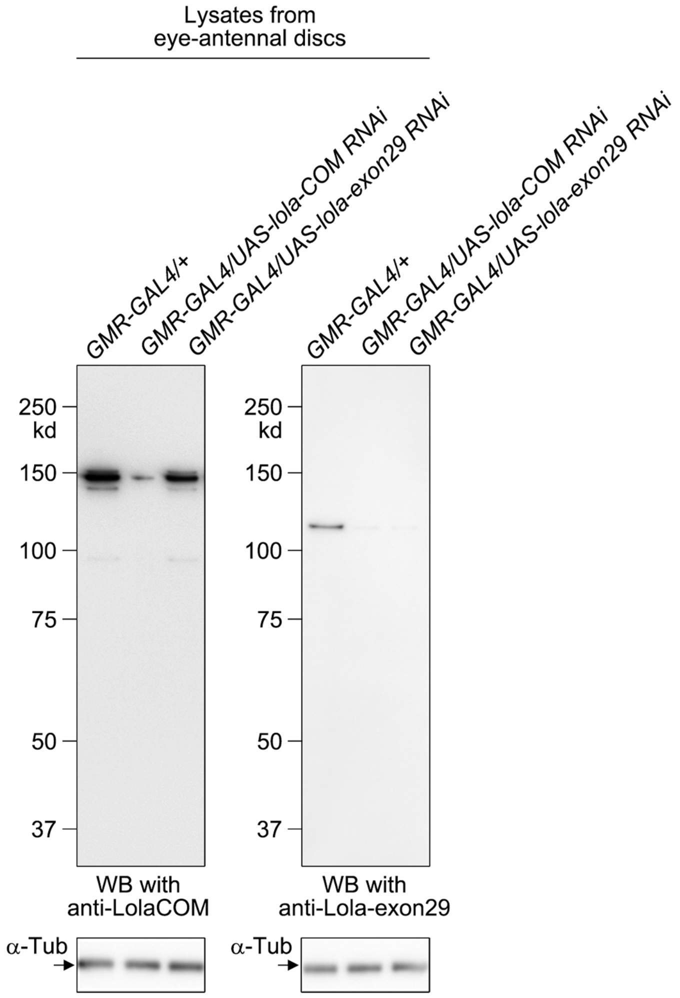

Supplementary Figure 6: Effectiveness of *lola* knockdown with RNAi evaluated by western blotting. *GMR-GAL4*-driven expression of *lola-COM RNAi* and *lola-exon 29 RNAi* reduced the amount of Lola proteins. Comparisons of a blot probed with the anti-LolaCOM antibody (left-hand panel) and that with anti-Lola-exon 29 antibody (right-hand panel) indicated the specific knockdown of Lola29M by *lola-exon 29 RNAi*. Source data are provided as a Source Data file.

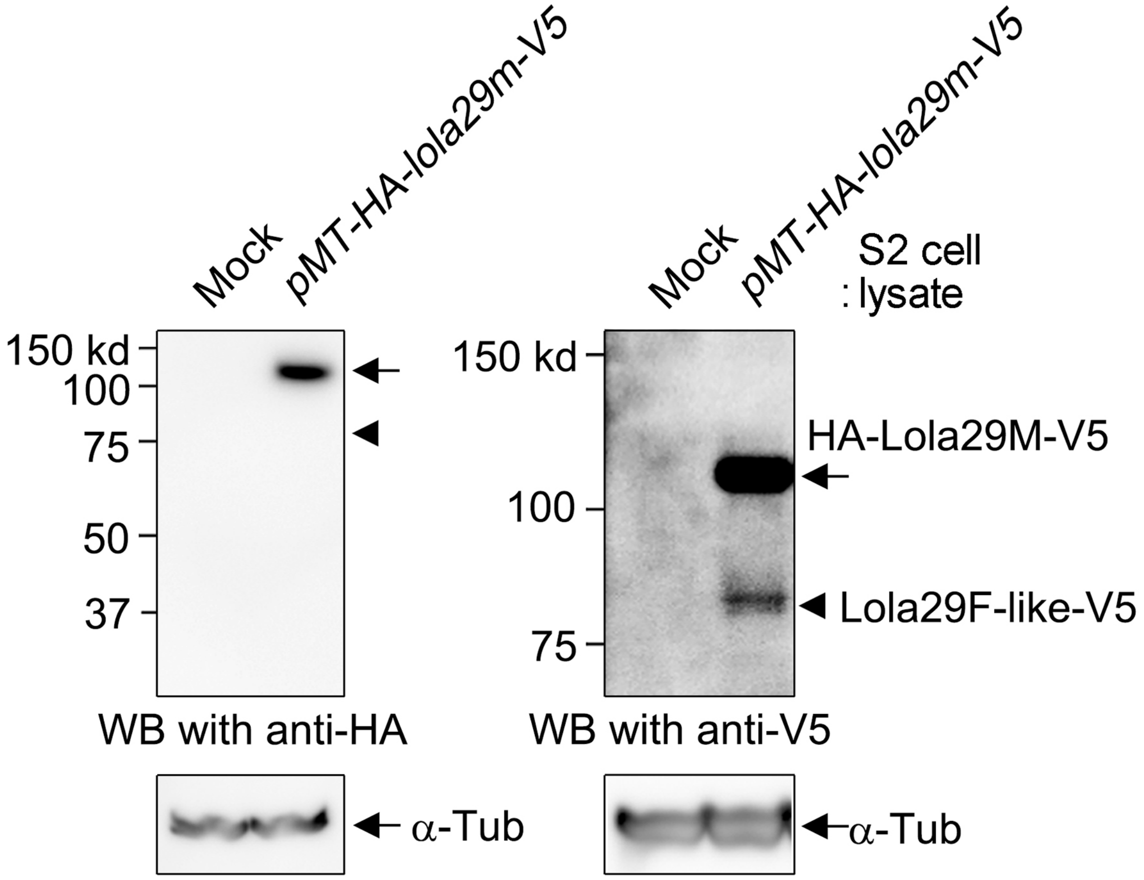

Supplementary Figure 7: S2 cells transfected with a full-length *lola29m* construct produce both Lola29M and Lola29F-like proteins.

Western blotting of lysates from S2 cells transfected with a vector encoding Lola29M decorated with the N-terminal HA-tag and the C-terminal V5-tag. Whereas an anti-V5 antibody detected two bands, Lola29M and Lola29F-like (right-hand side panel), an anti-HA antibody detected only Lola29M (left-hand side panel), suggesting that Lola29F-like may be an N-terminally truncated derivative of Lola29M. Source data are provided as a Source Data file.

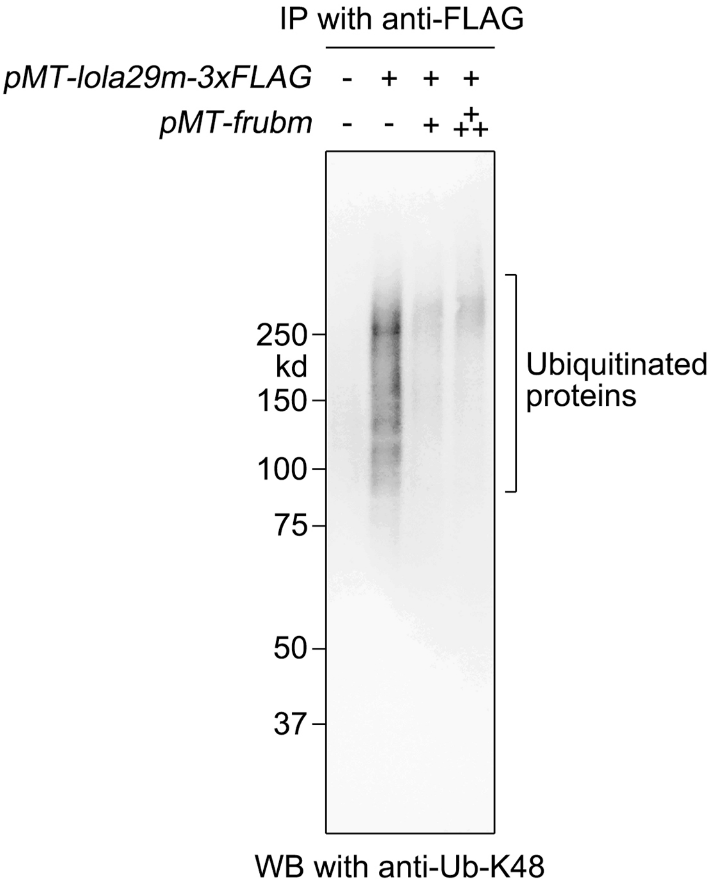

Supplementary Figure 8: Lola29M is K48-polyubiquitinated.

Western blotting of K48-polyubiquitinated proteins in immunoprecipitates of transfected S2 cells. The anti-Ub-K48 antibody was used as a probe in the western blotting. The proteins were immunoprecipitated with the anti-FLAG antibody that recognizes Lola29M::3xFLAG, which is overexpressed in S2 cells alone or together with FruBM. 0 (-) or 1 (+) µg of *pMT-lola29m-3xFLAG* and 0 (-), 1 (+) or 3 (++) µg of *pMT-frubm* were cotransfected into S2 cells (indicated above the gel). Source data are provided as a Source Data file.

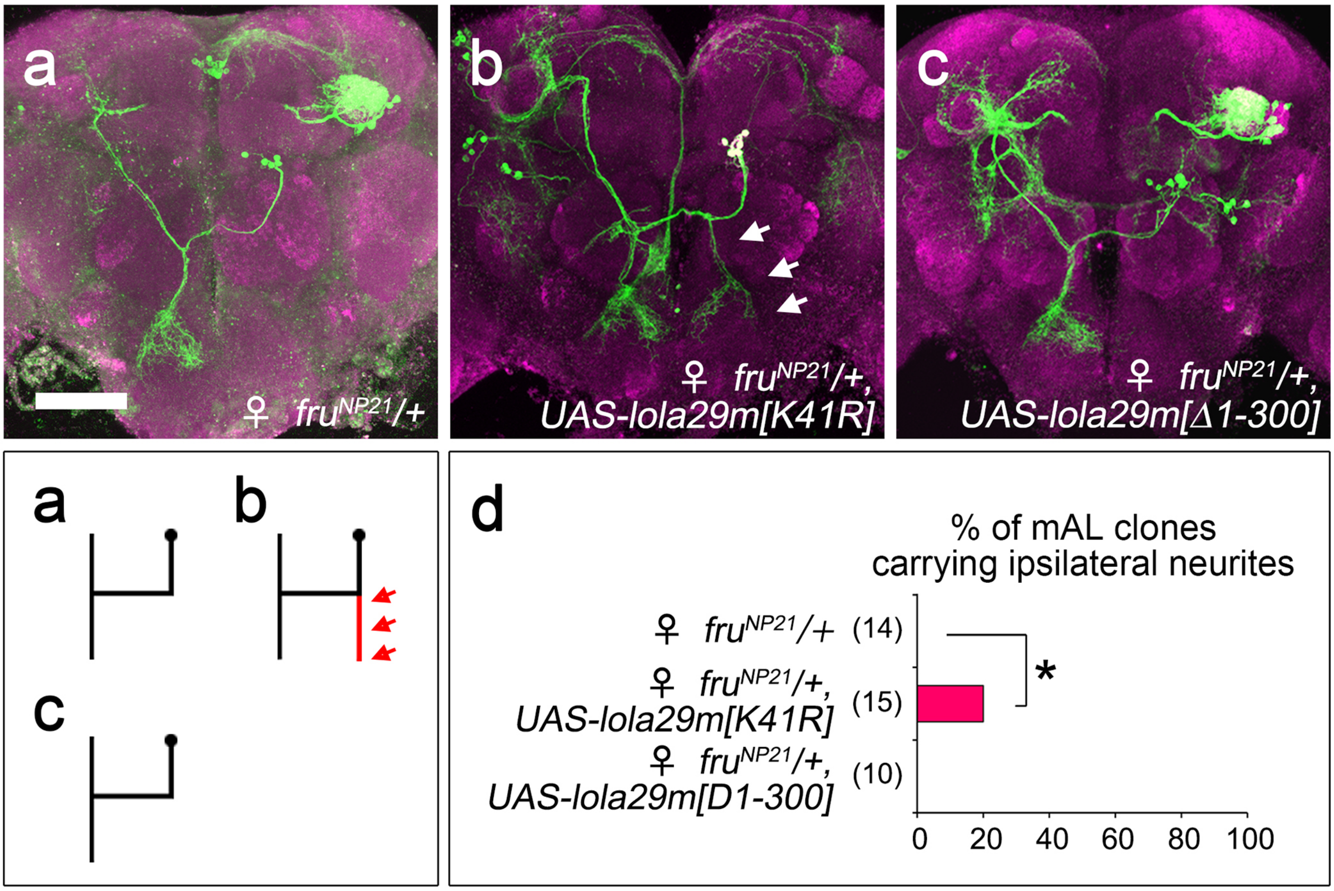

Supplementary Figure 9: Lola29M promotes the male-specific ipsilateral neurite formation in mAL neurons.

mAL neuroblast clones induced by MARCM in *fru*^{NP21} heterozygous females. Overexpression of truncation-resistant Lola29M[K41R] induced the ipsilateral neurite in some females (**b** cf. **a**), whereas overexpression of Lola29F-like (Lola29M[Δ1-300]) did not (**c**). **d**, Quantification of the effects of overexpression of Lola29M[K41R] and Lola29F-like (Lola29M[Δ1-300]) on the proportion of flies with the ipsilateral neurite. *: $P<0.05$ by the Fisher's exact probability test. Scale bar: 50 μm.

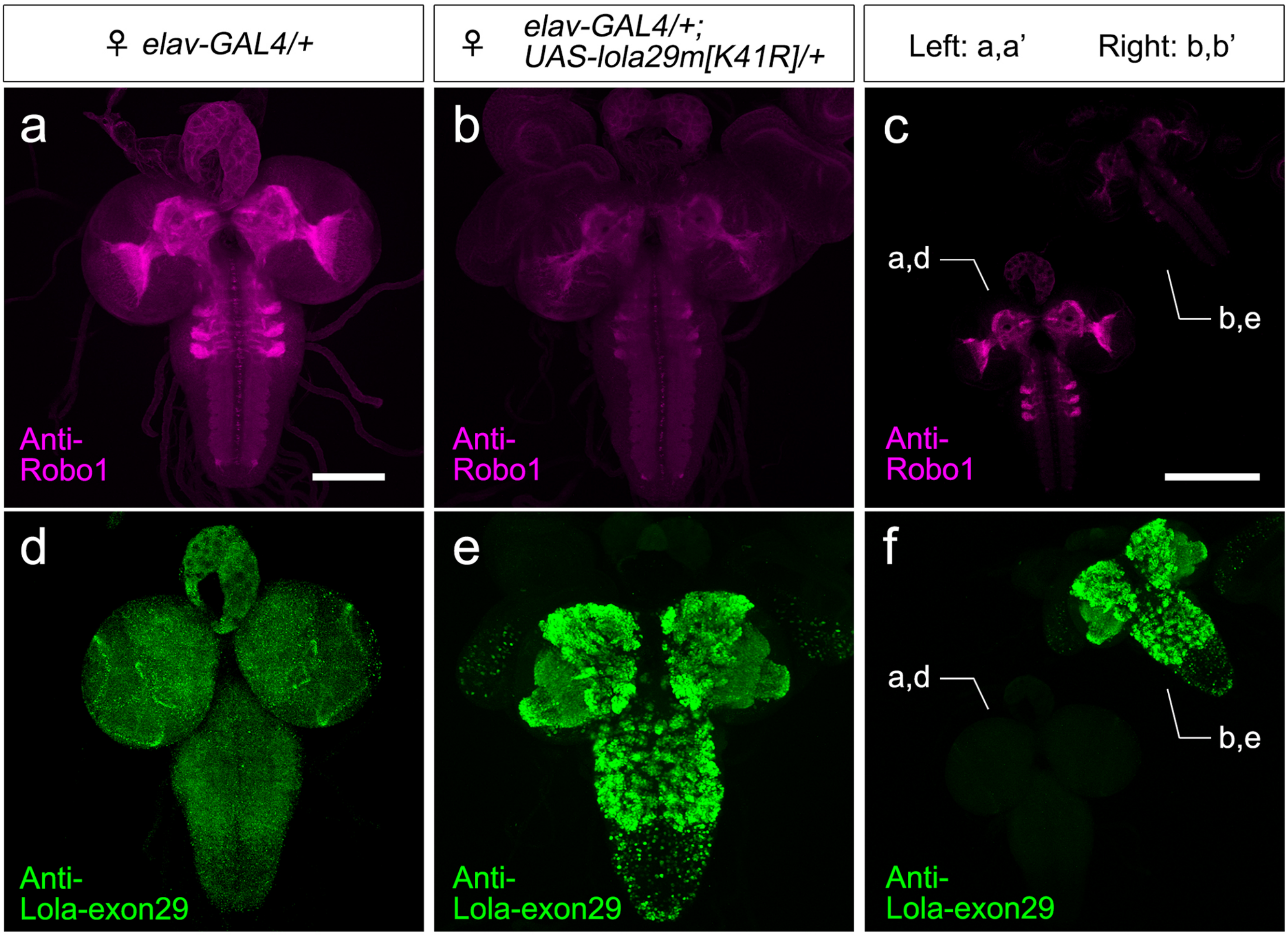

Supplementary Figure 10: Diminished Robo1 immunoreactivity of the larval CNS by Lola29M[K41R] overexpression.

The brain-VNC complexes from female third instar larvae with (**b**, **c**, **e**, **f**) or without (**a**, **c**, **d**, **f**) Lola29M[K41R] overexpression were subjected to the double immunostaining for Robo1 (**a-c**) and Lola29M/F (**d-f**). The control and test samples were processed in the same tubes at the same time. Scale bars: 100 μm in (**a, b, d, e**) and 200 μm in (**c, f**).

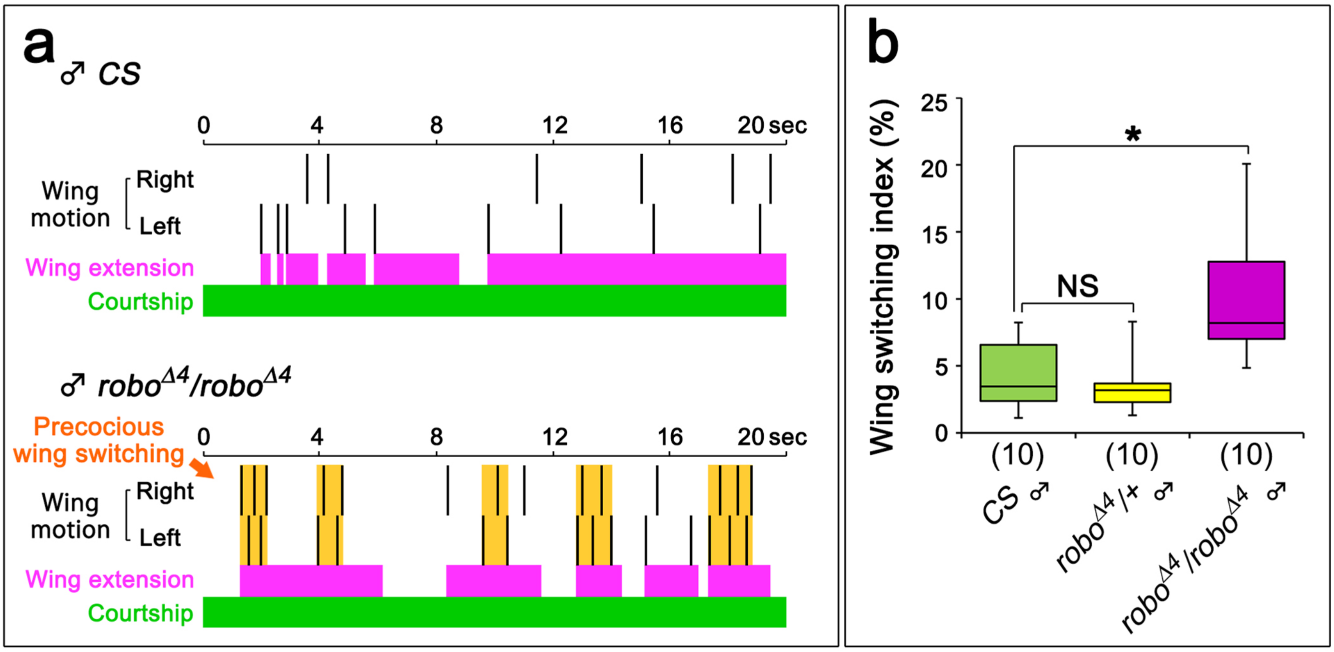

Supplementary Figure 11: Precocious wing switching during male courtship induced by the deletion of DR1 within the *robo1* promoter.

a, Examples of ethograms of a wild-type (CS) male and a *robo1*$^{\Delta4}$/*robo1*$^{\Delta4}$ mutant male. The time elapsed since the start of observation is shown on the top. The period during which the fly displayed precocious wing switching (orange bar), wing extension (magenta bar), or any courtship actions (green bar) is indicated. Vertical lines above the magenta bars indicate the time at which the fly switched the wing to be extended from the left wing to the right wing (right) and vice versa (left). **b**, The wing usage pattern in courtship compared among the indicated genotypes. The larger wing switching index indicates more-frequent switching of the right and left wings during courtship. The numbers of flies examined are indicated in parentheses below the abscissa. The box plot shows median and 10th, 25th, 75th, and 90th percentiles. The statistical differences among the datasets were evaluated by the Kruskal-Wallis analysis of variance followed by Steel's nonparametric multiple comparisons. *$p < 0.05$.

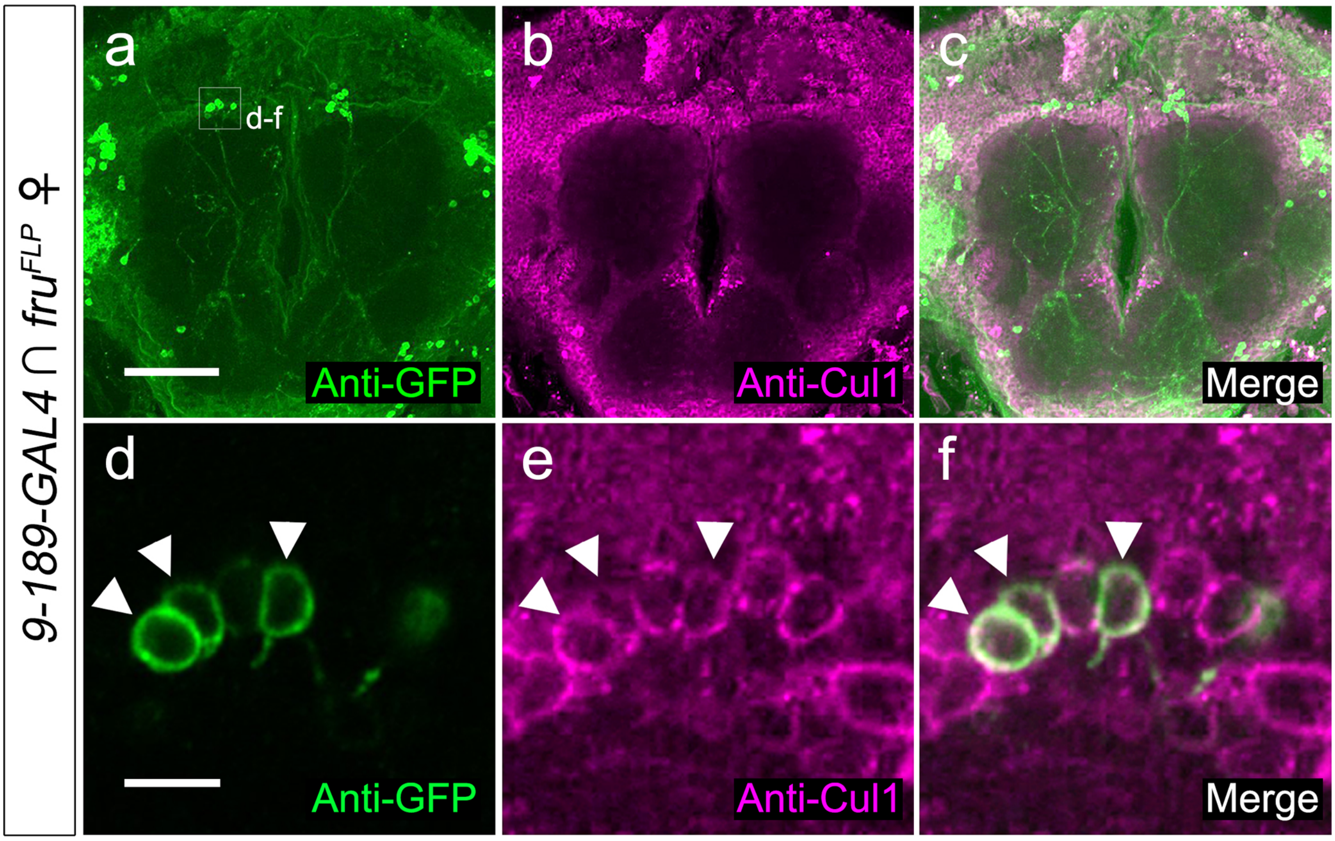

Supplementary Figure 12: Cul1 expression in mAL neurons.

A female brain doubly stained for GFP (**a**, **c**, **d**, **f**; green) and Cul1 (**b**, **c**, **e**, **f**; magenta) is shown at lower (**a-c**) and higher (**d-f**) magnification (scale bars indicate 50 μm for **a-c** and 5 μm for **d-f**). GFP expression was targeted to mAL neurons by the intersection of *9-189-GAL4* and *fru*^{FLP} (arrowheads).

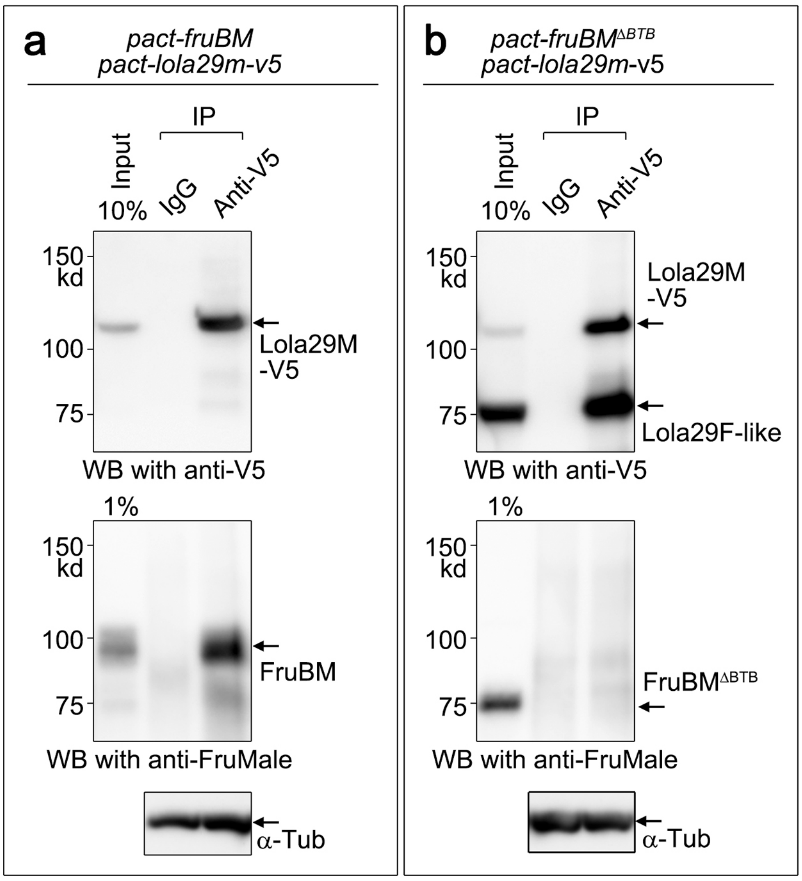

Supplementary Figure 13: Lola29M-FruBM interactions depend on the BTB domain of each.
The antibody that recognizes the C-terminal V5 tag of Lola29M-V5 (anti-V5) precipitated intact FruBM (**a**) but not BTB-deleted FruBM (FruBMΔBTB: **b**) in lysates from S2 cells cotransfected with constructs encoding the respective proteins. Note that the lack of the BTB domain of FruBM resulted in the production of Lola29F-like, which was not detected when both FruBM and Lola29M were structurally intact and had the BTB-domain. Source data are provided as a Source Data file.

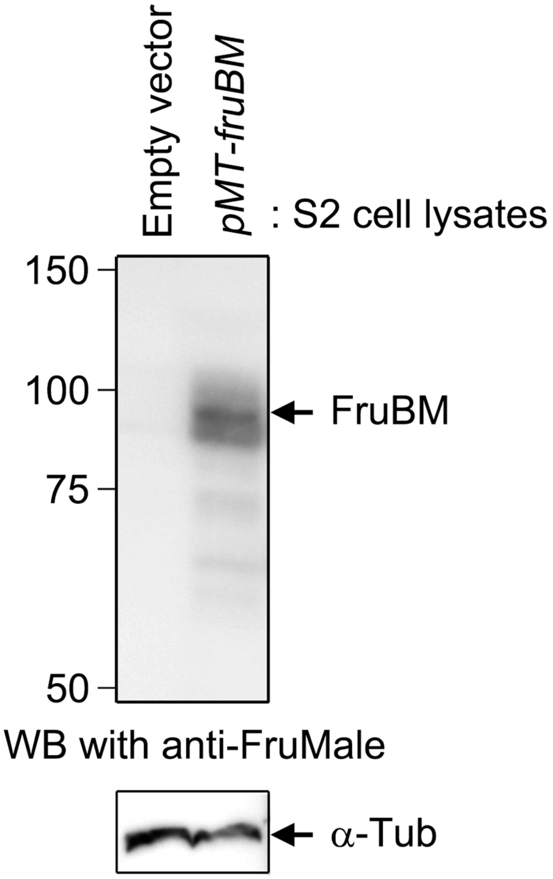

Supplementary Figure 14: Western Blot analysis of FruBM in S2 cell lysates.

FruBM protein was detected only when the cells were transfected with a FruBM-encoding sequence. α-tubulin served as an internal control. Source data are provided as a Source Data file.

Supplementary Table 1. List of proteins identified by mass spectrometry in immunoprecipitates that were obtained with an antibody recognizing Lola29M.

Accession	Description	Score	Coverage	# Peptides	# AAs	MW [kDa]
Q9V5M3	Longitudinals lacking protein, isoforms N/O/W/X/Y OS=Drosophila melanogaster GN=lola PE=1 SV=3 - [LOLA6_DROME]	3246.00	25.40	19	878	96.1
P02828	Heat shock protein 83 OS=Drosophila melanogaster GN=Hsp83 PE=1 SV=1 - [HSP83_DROME]	1464.02	55.23	39	717	81.8
P52034	ATP-dependent 6-phosphofructokinase OS=Drosophila melanogaster GN=Pfk PE=2 SV=2 - [PFKA_DROME]	1193.64	51.40	31	788	86.6
Q9VHP0	ATP-dependent RNA helicase bel OS=Drosophila melanogaster GN=bel PE=1 SV=1 - [DDX3_DROME]	1182.89	60.03	37	798	85.0
Q99323	Myosin heavy chain, non-muscle OS=Drosophila melanogaster GN=zip PE=1 SV=2 - [MYSN_DROME]	980.80	18.08	28	2057	236.5
P29844	Heat shock 70 kDa protein cognate 3 OS=Drosophila melanogaster GN=Hsc70-3 PE=2 SV=2 - [HSP7C_DROME]	813.14	38.57	22	656	72.2
P54351	Vesicle-fusing ATPase 2 OS=Drosophila melanogaster GN=Nsf2 PE=2 SV=2 - [NSF2_DROME]	703.21	52.39	31	752	83.4
P11147	Heat shock 70 kDa protein cognate 4 OS=Drosophila melanogaster GN=Hsc70-4 PE=1 SV=3 - [HSP7D_DROME]	689.32	41.32	26	651	71.1
Q9NFU0	Fragile X mental retardation syndrome-related protein 1 OS=Drosophila melanogaster GN=Fmr1 PE=1 SV=1 - [FMR1_DROME]	639.87	38.16	19	684	76.0
Q7KN90	Cysteine--tRNA ligase, cytoplasmic OS=Drosophila melanogaster GN=Aats-cys PE=1 SV=1 - [SYCC_DROME]	627.24	60.19	32	741	84.2
P10987	Actin-5C OS=Drosophila melanogaster GN=Act5C PE=1 SV=4 - [ACT1_DROME]	565.54	60.11	16	376	41.8
Q9XYU0	DNA replication licensing factor Mcm7 OS=Drosophila melanogaster GN=Mcm7 PE=1 SV=1 - [MCM7_DROME]	507.45	33.75	23	720	81.2
Q0E940	Eukaryotic translation initiation factor 3 subunit B OS=Drosophila melanogaster GN=eIF3-S9 PE=1 SV=1 - [EIF3B_DROME]	453.38	30.87	16	690	80.4
Q9VHR8	Dipeptidyl peptidase 3 OS=Drosophila melanogaster GN=DppIII PE=2 SV=2 - [DPP3_DROME]	449.97	27.74	21	786	89.1
O46037	Vinculin OS=Drosophila melanogaster GN=Vinc PE=1 SV=1 - [VINC_DROME]	445.56	20.29	14	961	106.2
Q9VNV3	ATP-dependent RNA helicase Ddx1 OS=Drosophila melanogaster GN=Ddx1 PE=2 SV=1 - [DDX1_DROME]	406.40	19.94	11	727	80.8
Q9W4X9	Centromere/kinetochore protein zw10 OS=Drosophila melanogaster GN=mit(1)15 PE=1 SV=2 - [ZW10_DROME]	401.08	30.51	15	721	82.2
Q94511	NADH-ubiquinone oxidoreductase 75 kDa subunit, mitochondrial OS=Drosophila melanogaster GN=ND75 PE=2 SV=3 - [NDUS1_DROME]	399.35	23.94	11	731	78.6
Q291L4	Cysteine--tRNA ligase, cytoplasmic OS=Drosophila pseudoobscura pseudoobscura GN=Aats-cys PE=3 SV=1 - [SYCC_DROPS]	391.27	19.97	12	741	83.7
P13469	DNA-binding protein modulo OS=Drosophila melanogaster GN=mod PE=1 SV=2 - [MODU_DROME]	373.82	30.26	12	542	60.3
Q9VRA2	Molybdenum cofactor sulfurase OS=Drosophila melanogaster GN=mal PE=1 SV=1 - [MOCOS_DROME]	349.64	19.33	10	781	88.0
Q9VFC8	Glycogen [starch] synthase OS=Drosophila melanogaster GN=GlyS PE=1 SV=2 - [GYS_DROME]	345.01	23.13	10	709	81.7
A1ZAX1	Eukaryotic translation initiation factor 3 subunit C OS=Drosophila melanogaster GN=eIF3-S8 PE=1 SV=1 - [EIF3C_DROME]	330.39	22.42	17	910	105.6
P29843	Heat shock 70 kDa protein cognate 1 OS=Drosophila melanogaster GN=Hsc70-1 PE=1 SV=1 - [HSP7A_DROME]	327.98	13.73	6	641	70.6
Q9VP61	Acetyl-coenzyme A synthetase OS=Drosophila melanogaster GN=AcCoAS PE=2 SV=1 - [ACSA_DROME]	315.81	31.49	16	670	75.9
Q960Z0	Kinesin-like protein Klp10A OS=Drosophila melanogaster GN=Klp10A PE=1 SV=1 - [KI10A_DROME]	313.98	22.61	14	805	88.6
P25991	Protein suppressor of forked OS=Drosophila melanogaster GN=su(f) PE=1 SV=2 - [SUF_DROME]	277.35	16.99	10	765	88.2
P10981	Actin-87E OS=Drosophila melanogaster GN=Act87E PE=1 SV=1 - [ACT5_DROME]	247.80	41.76	12	376	41.8
B4HY41	Elongation factor G, mitochondrial OS=Drosophila sechellia GN=ico PE=3 SV=1 - [EFGM_DROSE]	236.87	22.15	12	745	83.5
Q9VEZ5	Inhibitor of nuclear factor kappa-B kinase subunit beta OS=Drosophila melanogaster GN=ird5 PE=1 SV=2 - [IKKB_DROME]	229.55	13.85	9	751	86.3
Q24311	Cullin homolog 1 OS=Drosophila melanogaster GN=Cul1 PE=1 SV=2 - [CUL1_DROME]	209.28	20.67	15	774	89.5
P20480	Protein claret segregational OS=Drosophila melanogaster GN=ncd PE=1 SV=1 - [NCD_DROME]	206.23	23.00	13	700	77.4
Q9W1A2	N-alpha-acetyltransferase, 35 NatC auxiliary subunit homolog OS=Drosophila melanogaster GN=CG4065 PE=2 SV=1 - [NAA35_DROME]	182.93	10.97	6	784	89.1
Q9VZI3	Unc-112-related protein OS=Drosophila melanogaster GN=Fit1 PE=1 SV=1 - [UN112_DROME]	176.39	10.88	6	708	80.4
Q9V8K2	Exocyst complex component 3 OS=Drosophila melanogaster GN=sec6 PE=2 SV=2 - [EXOC3_DROME]	175.79	11.38	8	738	86.6
Q7KN62	Transitional endoplasmic reticulum ATPase TER94 OS=Drosophila melanogaster GN=TER94 PE=1 SV=1 - [TERA_DROME]	175.72	15.98	9	801	88.8
Q9VSH4	Cleavage and polyadenylation specificity factor subunit CG7185 OS=Drosophila melanogaster GN=CG7185 PE=1 SV=2 - [CPSF6_DROME]	172.77	8.90	4	652	71.1

Q04047	Protein no-on-transient A OS=Drosophila melanogaster GN=nonA PE=1 SV=2 - [NONA_DROME]	167.11	10.43	6	700	76.9
Q9Y0Y6	Nuclear receptor-binding protein homolog OS=Drosophila melanogaster GN=Madm PE=1 SV=1 - [NRBP_DROME]	161.59	16.01	7	637	70.5
P08736	Elongation factor 1-alpha 1 OS=Drosophila melanogaster GN=Ef1alpha48D PE=1 SV=2 - [EF1A1_DROME]	155.35	24.41	7	463	50.3
Q24560	Tubulin beta-1 chain OS=Drosophila melanogaster GN=betaTub56D PE=1 SV=2 - [TBB1_DROME]	154.62	23.49	8	447	50.1
Q7K4N3	Nuclear cap-binding protein subunit 1 OS=Drosophila melanogaster GN=Cbp80 PE=1 SV=1 - [NCBP1_DROME]	146.72	11.88	6	800	93.2
P13060	Elongation factor 2 OS=Drosophila melanogaster GN=EF2 PE=1 SV=4 - [EF2_DROME]	145.71	15.76	10	844	94.4
B3P211	Eukaryotic translation initiation factor 3 subunit A OS=Drosophila erecta GN=eIF3-S10 PE=3 SV=1 - [EIF3A_DROER]	138.47	5.46	5	1135	133.5
Q24151	Signal transducer and transcription activator OS=Drosophila melanogaster GN=Stat92E PE=1 SV=1 - [STAT_DROME]	134.96	9.72	6	761	86.4
Q9V877	Kinesin-like protein subito OS=Drosophila melanogaster GN=sub PE=1 SV=1 - [SUB_DROME]	134.73	14.49	7	628	71.3
Q9XYU1	DNA replication licensing factor Mcm3 OS=Drosophila melanogaster GN=Mcm3 PE=1 SV=1 - [MCM3_DROME]	134.17	7.20	4	819	90.9
P46150	Moesin/ezrin/radixin homolog 1 OS=Drosophila melanogaster GN=Moe PE=1 SV=2 - [MOEH_DROME]	131.81	11.25	6	578	68.1
Q9I7K6	Protein NASP homolog OS=Drosophila melanogaster GN=CG8223 PE=1 SV=1 - [NASP_DROME]	130.68	5.69	2	492	51.9
P06603	Tubulin alpha-1 chain OS=Drosophila melanogaster GN=alphaTub84B PE=2 SV=1 - [TBA1_DROME]	120.94	12.89	4	450	49.9
Q9VYA0	Probable glutamine-dependent NAD(+) synthetase OS=Drosophila melanogaster GN=CG9940 PE=1 SV=1 - [NADE_DROME]	120.11	12.58	6	787	87.6
Q9VJZ7	Ribosomal RNA processing protein 1 homolog OS=Drosophila melanogaster GN=Nnp-1 PE=1 SV=1 - [RRP1L_DROME]	111.46	12.08	6	687	78.9
Q95RI5	Failed axon connections OS=Drosophila melanogaster GN=fax PE=1 SV=1 - [FAXC_DROME]	109.95	7.42	2	418	47.1
P22700	Calcium-transporting ATPase sarcoplasmic/endoplasmic reticulum type OS=Drosophila melanogaster GN=Ca-P60A PE=1 SV=2 - [ATC1_DROME]	108.83	5.10	4	1020	111.6
Q9VUY0	MOXD1 homolog 1 OS=Drosophila melanogaster GN=CG5235 PE=2 SV=2 - [MOX11_DROME]	105.38	8.74	5	698	79.4
Q8T4F7	Protein enabled OS=Drosophila melanogaster GN=ena PE=1 SV=4 - [ENA_DROME]	102.04	5.20	4	980	104.8
P35600	Replication factor C subunit 1 OS=Drosophila melanogaster GN=Gnf1 PE=1 SV=2 - [RFC1_DROME]	101.27	4.87	4	986	108.5
B4HW93	Pescadillo homolog OS=Drosophila sechellia GN=GM12330 PE=3 SV=1 - [PESC_DROSE]	100.93	9.09	5	627	73.8
P16371	Protein groucho OS=Drosophila melanogaster GN=gro PE=1 SV=3 - [GROU_DROME]	99.29	12.19	5	730	80.2
Q9VD51	Probable ATP-dependent RNA helicase pitchoune OS=Drosophila melanogaster GN=pit PE=2 SV=2 - [DDX18_DROME]	95.52	10.59	6	680	76.9
Q7PLI7	Serine/threonine-protein kinase CG17528 OS=Drosophila melanogaster GN=CG17528 PE=1 SV=1 - [DCLK_DROME]	94.66	4.41	3	748	82.9
Q9VI55	E3 UFM1-protein ligase 1 homolog OS=Drosophila melanogaster GN=CG1104 PE=1 SV=1 - [UFL1_DROME]	91.80	11.51	6	782	87.4
P17886	Protein crooked neck OS=Drosophila melanogaster GN=crn PE=2 SV=2 - [CRN_DROME]	91.02	7.83	5	702	84.2
P48591	Ribonucleoside-diphosphate reductase large subunit OS=Drosophila melanogaster GN=RnrL PE=1 SV=2 - [RIR1_DROME]	90.85	8.37	7	812	91.9
P16620	Tyrosine-protein phosphatase 69D OS=Drosophila melanogaster GN=Ptp69D PE=1 SV=2 - [PTP69_DROME]	83.33	3.15	4	1462	167.4
Q7K4B3	Probable elongator complex protein 2 OS=Drosophila melanogaster GN=Elp2 PE=1 SV=1 - [ELP2_DROME]	80.64	1.89	1	794	88.9
Q7K4Q5	Probable protein phosphatase CG10417 OS=Drosophila melanogaster GN=CG10417 PE=1 SV=1 - [Y0417_DROME]	80.38	9.06	4	662	72.3
Q95ZE8	60S ribosomal protein L14 OS=Drosophila virilis GN=RpL14 PE=3 SV=1 - [RL14_DROVI]	77.38	7.50	1	160	18.7
P32865	G protein-coupled receptor kinase 1 OS=Drosophila melanogaster GN=Gprk1 PE=2 SV=2 - [GPRK1_DROME]	75.27	3.29	2	700	80.5
Q8MKW7	Ribonuclease Z, mitochondrial OS=Drosophila melanogaster GN=JhI-1 PE=1 SV=2 - [RNZ_DROME]	74.92	4.70	3	766	85.4
P34739	Transcription termination factor 2 OS=Drosophila melanogaster GN=lds PE=1 SV=2 - [TTF2_DROME]	68.72	5.09	5	1061	118.3
Q95RJ9	F-box-like/WD repeat-containing protein ebi OS=Drosophila melanogaster GN=ebi PE=1 SV=2 - [EBI_DROME]	68.43	6.57	5	700	72.3
Q24323	Semaphorin-2A OS=Drosophila melanogaster GN=Sema-2a PE=1 SV=2 - [SEM2A_DROME]	67.67	3.31	2	724	82.9
Q9NBD7	CLIP-associating protein OS=Drosophila melanogaster GN=chb PE=1 SV=1 - [CLASP_DROME]	67.04	4.90	5	1491	165.5
Q9W4M9	tRNA (cytosine(34)-C(5))-methyltransferase OS=Drosophila melanogaster GN=Nsun2 PE=2 SV=1 - [NSUN2_DROME]	64.33	4.16	2	746	84.1
Q9NJH0	Elongation factor 1-gamma OS=Drosophila melanogaster GN=Ef1gamma PE=2 SV=2 - [EF1G_DROME]	60.38	3.02	1	431	48.9
Q8IGJ0	Protein EFR3 homolog cmp44E OS=Drosophila melanogaster GN=stmA PE=2 SV=3 - [EFR3_DROME]	57.46	2.40	2	834	93.9
Q8IPK4	Glycosyltransferase 25 family member OS=Drosophila melanogaster GN=CG31915 PE=2 SV=1 - [GLT25_DROME]	55.39	11.11	6	612	71.1

Q9VS46	RINT1-like protein OS=Drosophila melanogaster GN=CG8605 PE=2 SV=2 - [RINT1_DROME]	54.50	2.90	2	724	83.5
Q9W1J3	Gastrulation defective protein 1 homolog OS=Drosophila melanogaster GN=CG5543 PE=2 SV=1 - [GAD1_DROME]	50.40	4.12	2	655	73.6
Q8MT06	Guanine nucleotide-binding protein-like 3 homolog OS=Drosophila melanogaster GN=Ns1 PE=1 SV=2 - [GNL3_DROME]	48.17	7.23	3	581	65.9
O62621	Coatomer subunit beta' OS=Drosophila melanogaster GN=beta'Cop PE=2 SV=2 - [COPB2_DROME]	42.38	2.95	2	914	102.6
O16797	60S ribosomal protein L3 OS=Drosophila melanogaster GN=RpL3 PE=1 SV=3 - [RL3_DROME]	42.32	6.73	2	416	46.9
Q8MQW8	Protein sprint OS=Drosophila melanogaster GN=spri PE=2 SV=3 - [SPRI_DROME]	41.45	1.17	2	1789	193.2
P35381	ATP synthase subunit alpha, mitochondrial OS=Drosophila melanogaster GN=blw PE=1 SV=2 - [ATPA_DROME]	40.74	2.17	1	552	59.4
Q9VLK2	Ribosomal L1 domain-containing protein CG13096 OS=Drosophila melanogaster GN=CG13096 PE=1 SV=1 - [Y3096_DROME]	39.13	5.58	2	681	74.3
P40417	Mitogen-activated protein kinase ERK-A OS=Drosophila melanogaster GN=rl PE=1 SV=3 - [ERKA_DROME]	38.79	2.13	1	376	43.1
Q9VAW5	La-related protein 1 OS=Drosophila melanogaster GN=larp PE=1 SV=5 - [LARP_DROME]	38.34	1.73	3	1673	178.0
Q9V3P0	Peroxiredoxin 1 OS=Drosophila melanogaster GN=Jafrac1 PE=1 SV=1 - [PRDX1_DROME]	38.27	4.12	1	194	21.7
P0CG69	Polyubiquitin OS=Drosophila melanogaster GN=Ubi-p63E PE=3 SV=1 - [UBIQP_DROME]	37.80	19.66	2	763	85.7
P83097	Putative tyrosine-protein kinase Wsck OS=Drosophila melanogaster GN=Wsck PE=2 SV=2 - [WSCK_DROME]	35.95	2.91	1	791	88.5
Q9W3K5	Glutamate--cysteine ligase OS=Drosophila melanogaster GN=Gclc PE=2 SV=1 - [GSH1_DROME]	35.01	3.35	2	717	80.7
Q9Y169	Exostosin-2 OS=Drosophila melanogaster GN=Ext2 PE=1 SV=1 - [EXT2_DROME]	34.94	1.81	1	717	82.7
Q9VJD3	Conserved oligomeric Golgi complex subunit 5 OS=Drosophila melanogaster GN=fws PE=2 SV=1 - [COG5_DROME]	34.80	3.33	2	751	84.9
P84236	Histone H3 OS=Drosophila hydei GN=His3 PE=3 SV=2 - [H3_DROHY]	33.51	5.15	1	136	15.4
P84052	Histone H2A OS=Drosophila erecta GN=His2A PE=3 SV=2 - [H2A_DROER]	33.24	7.26	1	124	13.4
Q9VT65	Calpain-B OS=Drosophila melanogaster GN=CalpB PE=1 SV=2 - [CANB_DROME]	32.92	1.19	1	925	103.7
P91679	Peptide transporter family 1 OS=Drosophila melanogaster GN=yin PE=1 SV=2 - [PEPT1_DROME]	32.57	3.90	2	743	82.2
Q9VF78	Conserved oligomeric Golgi complex subunit 2 OS=Drosophila melanogaster GN=ldlCp PE=2 SV=1 - [COG2_DROME]	31.14	1.27	1	710	81.1
Q06559	40S ribosomal protein S3 OS=Drosophila melanogaster GN=RpS3 PE=1 SV=1 - [RS3_DROME]	30.51	3.66	1	246	27.5
Q02748	Eukaryotic initiation factor 4A OS=Drosophila melanogaster GN=eIF-4a PE=1 SV=3 - [IF4A_DROME]	30.51	2.48	1	403	45.8
Q07407	Fibroblast growth factor receptor homolog 1 OS=Drosophila melanogaster GN=htl PE=1 SV=3 - [FGFR1_DROME]	30.16	1.92	1	729	82.5
Q29C43	Neutral ceramidase OS=Drosophila pseudoobscura pseudoobscura GN=CDase PE=3 SV=1 - [NCASE_DROPS]	29.72	3.69	2	704	78.0
O01346	Beta-1,4-mannosyltransferase egh OS=Drosophila melanogaster GN=egh PE=2 SV=1 - [EGH_DROME]	28.94	2.63	1	457	52.0
P29742	Clathrin heavy chain OS=Drosophila melanogaster GN=Chc PE=1 SV=1 - [CLH_DROME]	26.72	1.43	2	1678	191.1
Q9V8Y7	Odorant receptor 56a OS=Drosophila melanogaster GN=Or56a PE=1 SV=2 - [OR56A_DROME]	26.64	1.43	1	419	48.9
P53625	Mannosyl-oligosaccharide alpha-1,2-mannosidase isoform B OS=Drosophila melanogaster GN=alpha-Man-I PE=2 SV=2 - [MA122_DROME]	26.54	1.24	1	643	72.5
Q9V9K7	Serrate RNA effector molecule homolog OS=Drosophila melanogaster GN=Ars2 PE=1 SV=2 - [SRRT_DROME]	26.27	2.23	2	943	107.2
Q9VC27	Nicastrin OS=Drosophila melanogaster GN=nct PE=1 SV=3 - [NICA_DROME]	25.81	2.45	1	695	77.9
Q9VAQ7	Pre-mRNA-splicing factor Slu7 OS=Drosophila melanogaster GN=Slu7 PE=1 SV=2 - [SLU7_DROME]	24.97	2.61	1	574	65.9
B4IXJ2	Protein cueball OS=Drosophila grimshawi GN=cue PE=3 SV=1 - [CUE_DROGR]	24.92	1.65	1	605	68.3
P17271	Histone H2B OS=Drosophila hydei GN=His2B PE=3 SV=2 - [H2B_DROHY]	24.01	7.32	1	123	13.7
P28668	Bifunctional glutamate/proline--tRNA ligase OS=Drosophila melanogaster GN=Aats-glupro PE=1 SV=2 - [SYEP_DROME]	23.77	0.53	1	1714	189.3
Q9V4W1	Nucleoporin GLE1 OS=Drosophila melanogaster GN=GLE1 PE=2 SV=1 - [GLE1_DROME]	23.49	2.51	2	677	77.3
Q9V411	Probable nucleolar GTP-binding protein 1 OS=Drosophila melanogaster GN=CG8801 PE=2 SV=1 - [NOG1_DROME]	23.08	1.53	1	652	75.3
Q9VSJ8	Exocyst complex component 7 OS=Drosophila melanogaster GN=exo70 PE=1 SV=2 - [EXOC7_DROME]	23.07	1.15	1	693	80.0
P27864	Recombination repair protein 1 OS=Drosophila melanogaster GN=Rrp1 PE=1 SV=2 - [RRP1_DROME]	22.61	1.18	1	679	74.6
P50887	60S ribosomal protein L22 OS=Drosophila melanogaster GN=RpL22 PE=1 SV=2 - [RL22_DROME]	21.56	4.01	1	299	30.6
Q24478	Centrosome-associated zinc finger protein CP190 OS=Drosophila melanogaster GN=Cp190 PE=1 SV=2 - [CP190_DROME]	20.66	0.64	1	1096	121.6

Q9XTM1	Exocyst complex component 5 OS=Drosophila melanogaster GN=sec10 PE=2 SV=1 - [EXOC5_DROME]	20.61	1.41	1	710	82.0
Q27415	Nucleoplasmin-like protein OS=Drosophila melanogaster GN=Nlp PE=1 SV=1 - [NLP_DROME]	20.52	7.89	1	152	17.0

The name of identified proteins (Description, 2nd column) is listed in order from largest to smallest peptide probability score (Score: 3rd column). Accession, accession number in NCBI; Coverage, the percent sequence coverage identified from MS/MS results; # Peptide, number of the identified peptides by LC-MS/MS; AAs, number of amino acids; MW, molecular weight. The S2 cells transfected with a Lola29M[Δ1-150] expression vector were the source of lysates subjected to immunoprecipitation. The E3 ubiquitin ligase Cullin1 is highlighted in red.

Supplementary Table 2. List of primer pairs for constructing plasmid vectors.

#	Name	Sequence 5'-3'	Plasmid vector	Note
1	fruBM-DBTB F1	TGCGAATTCGGATCCCAACAT	*pact-FLAG-fruBM[DBTB]* and *pact-FLAG-fruBM[DZn-finger,DBTB]* vectors	Paired with FruBM-DBTB R1
2	fruBM-DBTB R1	GTCCATCGCTCCTTGGTCAG	*pact-FLAG-fruBM[DBTB]* and *pact-FLAG-fruBM[DZn-finger,DBTB]* vectors	
3	fruBM-DBTB F2	CAAGGAGCGATGGACACAGATAACAACAATCTGAA	*pact-FLAG-fruBM[DBTB]* and *pact-FLAG-fruBM[DZn-finger,DBTB]* vectors	Paired with FruBM-DBTB R2
4	fruBM-DBTB R2	TCTTCATGGTCGACGGTACCTTAAATGGATGAGTTCAGCT	*pact-FLAG-fruBM[DBTB]* and *pact-FLAG-fruBM[DZn-finger,DBTB]* vectors	
5	HA-lola29m(WT) F	GGGGTACCCAAAATGTACCCCTACGATGTGCCCGATTACGCCGATGACGATCAGCAGTTTTGTTTGC (KpnI)	*pMT-HA-lola29m-V5* vector	Paired with HA-lola29m(WT) R
6	HA-lola29m(WT) R	GGGCGGCCGCGGTTGCAAGTATTGCCTCCCC (NotI)	*pMT-HA-lola29m-V5* vector	
7	COM F	GGGATCTAGATCGGGGTACC	*pMT-HA-lola29m[K41R]-V5* vector	Paired with COM R
8	COM R	TGACCTCGAGCGGCCGCGGTTG	*pMT-HA-lola29m[K41R]-V5* vector	
9	K41R R	GAGAAAGCGGCCCTCGGCGG	*pMT-HA-lola29m[K41R]-V5* vector	Paired with COM F
10	K41R F	GAGGGCCGCTTTCTCAAGGCCCA	*pMT-HA-lola29m[K41R]-V5* vector	Paired with COM R
11	K44R R	GTGGGCGCGGAGAAATTTGC	*pMT-HA-lola29m[K44R]-V5* vector	Paired with COM F
12	K44R F	TTTCTCCGCGCCCACAAGGTG	*pMT-HA-lola29m[K44R]-V5* vector	Paired with COM R
13	K47R R	GCACCACGCGGTGGGCCTTG	*pMT-HA-lola29m[K47R]-V5* vector	Paired with COM F
14	K47R F	CCCACCGCGTGGTGCTGTCA	*pMT-HA-lola29m[K47R]-V5* vector	Paired with COM R
15	K67R R	ATGGGATGGCGGTCGTACTG	*pMT-HA-lola29m[K67R]-V5* vector	Paired with COM F
16	K67R F	CGACCGCCATCCCATCTTTA	*pMT-HA-lola29m[K67R]-V5* vector	Paired with COM R
17	K74R R	TGACATCGCGGAGTATAAAG	*pMT-HA-lola29m[K74R]-V5* vector	Paired with COM F
18	K74R F	TACTCCGCGATGTCAAGTAC	*pMT-HA-lola29m[K74R]-V5* vector	Paired with COM R
19	K77R R	CTTGGTAGCGGACATCCTTG	*pMT-HA-lola29m[K77R]-V5* vector	Paired with COM F
20	K77R F	ATGTCCGCTACCAAGAGCTG	*pMT-HA-lola29m[K77R]-V5* vector	Paired with COM R
21	K105R R	TCGGCGGCGCGGAGCAGAGC	*pMT-HA-lola29m[K105R]-V5* vector	Paired with COM F
22	K105R F	GCTCCGCGCCGCCGAATCGC	*pMT-HA-lola29m[K105R]-V5* vector	Paired with COM R
23	K113R R	GAAAGGCCGCGGATCTGAAG	*pMT-HA-lola29m[K113R]-V5* vector	Paired with COM F
24	K113R F	GATCCGCGGCCTTTCGGACA	*pMT-HA-lola29m[K113R]-V5* vector	Paired with COM R
25	K127R R	GACTCTGGGCGGGGAGCTAC	*pMT-HA-lola29m[K127R]-V5* vector	Paired with COM F
26	K127R F	TCCCCGCCCAGAGTCCTCCG	*pMT-HA-lola29m[K127R]-V5* vector	Paired with COM R
27	K138R R	CTCAGGCGACCGCCGCGATG	*pMT-HA-lola29m[K138R]-V5* vector	Paired with COM F
28	K138R F	CGGCGGTCGCCTGAGCGGTG	*pMT-HA-lola29m[K138R]-V5* vector	Paired with COM R
29	K149R R	CGAGCCCGGCGAGTTTGCTC	*pMT-HA-lola29m[K149R]-V5* vector	Paired with COM F
30	K149R F	AACTCGCCGGGCTCGACTGG	*pMT-HA-lola29m[K149R]-V5* vector	Paired with COM R
31	K181R R	CGTCGGACGCGTCGGCGACG	*pMT-HA-lola29m[K181R]-V5* vector	Paired with COM F
32	K181R F	CCGACGCGTCCGACGTCGCA	*pMT-HA-lola29m[K181R]-V5* vector	Paired with COM R
33	K255R R	CAGTCTTGCGGGTAACGTTG	*pMT-HA-lola29m[K255R]-V5* vector	Paired with COM F
34	K255R F	TTACCCGCAAGACTGAAAGC	*pMT-HA-lola29m[K255R]-V5* vector	Paired with COM R
35	K256R R	CAGTGCGTTTGGTAACGTTG	*pMT-HA-lola29m[K256R]-V5* vector	Paired with COM F
36	K256R F	TTACCAAACGCACTGAAAGCGCT	*pMT-HA-lola29m[K256R]-V5* vector	Paired with COM R
37	K261R R	GATGTTAGGCGAGCGCTTTC	*pMT-HA-lola29m[K261R]-V5* vector	Paired with COM F
38	K261R F	CGCTCGCCTAACATCCTCGAC	*pMT-HA-lola29m[K261R]-V5* vector	Paired with COM R

39	FLAG-lola29m F	GGGCGGCCGCCAAAATGGATTACAAGGATGACGATGACAAGGATGACGATCAGCAGTTTTGTTTGC (NotI)	*pact-FLAG-lola29m* and *pact-FLAG-lola29m[K41R]* vectors	Paired with FLAG-lola29m R
40	FLAG-lola29m R	GGGGTACCCTAGGTTGCAAGTATTGCCTCCCC (KpnI)	*pact-FLAG-lola29m* and *pact-FLAG-lola29m[K41R]* vectors	
41	FLAG-lola29m[D1-300]	GGGCGGCCGCCAAAATGGATTACAAGGATGACGATGACAAG ACAAGCGATGCCATTAACACCG (NotI)	*pact-FLAG-lola29m[D1-300]* vector	Paired with FLAG-lola29m R
42	pJFRC-lola29m F	GGCCGCGGCTCGAGGGTACCAACTTAAAAAAAAAAATCAAAATGTACCCCTACGATGTGCCCGAT	*pJFRC81-HA-lola29m-V5* , *pJFRC81-HA-lola29m[K41R]-V5* and *pJFRC81-HA-lola29m[D1-300]-V5* vectors	Paired with pJFRC-lola29m R
43	pJFRC-lola29m R	AACGATTCATTCTAGATCAATGGTGATGGTGATGATGACC	*pJFRC81-HA-lola29m-V5, pJFRC81-HA-lola29m[K41R]-V5 and pJFRC81-HA-lola29m[D1-300]-V5 vectors*	
44	pact-lola29m F	TCCTCTAGAGCGGCCGCCAAAATGTACCCCTACGATG	*pact-ha-lola29m-v5 and pact-ha-lola29m[D1-118]-v5 vectors*	Paired with pact-lola29m R
45	pact-lola29m R	TCTTCATGGTCGACGGTACCTCAATGGTGATGGTGATGAT	*pact-ha-lola29m-v5 and pact-ha-lola29m[D1-118]-v5 vectors*	
46	pGL3-promoter F	TTTCTCTATCGATAGGTACC	*-0.9 kb robo1 luciferase-reporter DDR1*	Paired with DDR1 R
47	DDR1 R	TTGGAAATTAACTATGATTCACGGCGCAGCGAAAA	*-0.9 kb robo1 luciferase-reporter DDR1*	
48	DDR1 F	ATAGTTAATTTCCAACAGTT	*-0.9 kb robo1 luciferase-reporter DDR1*	Paired with pGL3-promoter R
49	pGL3-promoter R	TGCAGATCGCAGATCTGCCT	*-0.9 kb robo1 luciferase-reporter DDR1*	
50	3xFLAG F	GGGATCTAGATCGGGGTACCCAAAATGTACCCATACGATGTTCCAGATTACGCTGACTACAAAGACGATGACGACAAGGACTACAAAGACGATGACGACAAGGACTACAAAGACGATGACGACAAGGATGACGATCAGCAGTTTTG	*pMT-3xFLAG-lola29m[K41R]* vector	Paired with lola29m R
51	3xFLAG D1-263 F	GGGATCTAGATCGGGGTACCCAAAATGTACCCATACGATGTTCCAGATTACGCTGACTACAAAGACGATGACGACAAGGACTACAAAGACGATGACGACAAGGACTACAAAGACGATGACGACAAGTCCTCGACAGCCGCCCCAGC	*pMT-3xFLAG-lola29m[D1-263]* vector	Paired with lola29m R
52	pJFRC-lola29m 6xV5 F	AACGATTCATTCTAGATCAATGGTGATGGTGATGATGACCGGTACGCGTAGAATCGAGACCGAGGAGAGGGTTAGGGATAGGCTTACCTTCGAACCGCGGGCCCTCTAGACTCGAGCGGCCCGTAGAATCGAGACCGAGGAGAGGGTTAGGGATAGGCTTACCGGCCGCCGTAGAATCGAGACCGAGGAGAGGGTTAGGGATAGGCTTACCGGCCGCCGTAGAATCGAGACCGAGGAGAGGGTTAGGGATAGGCTTACCGGCCGCCGTAGAATCGAGACCGAGGAGAGGGTTAGGGATAGGCTTACCACCGGTACGCGTAGAATCGAGACCGAGGAGAGGGTTAGGGATAGGCTTACCTTCGAATGGGTGACCTCGAGCGGCCGCGGTTGCAAGTATTGCCTCCC	*pJFRC81-HA-lola29m-6xV5* vector	Paired with pJFRC-lola29m F

Single underlining is used to denote 15 bp overlaps for In-fusion cloning. Double underlining is used to denote restriction enzyme recognition sites. An amino acid replacement (K to R) is highlighted in red. F, forward primer; R, reverse primer.

Supplementary Table 3. List of primer pairs for 5'/3' RACE experiments (Supplementary Fig. 5).

#	Name	Sequence 5'-3'	Note	
1	5' RACE primer (F)	Mixture of oligos: 5'-CTAATACGACTCACTATAGGGCAAGCAGTGGTAT CAACGCAGAGT-3' (0.4 μM) and 5'-CTAATACGACTCACTATAGGGC-3' (2 μM)	Same as Universal Primer A Mix (UPM) in SMARTer RACE cDNA Amplification Kit (Clontech)	Paired with Primer 1
2	Primer 1 (R)	CGTGCTGGTCAACCTTCATGGCCTCC		
3	Primer 2 (F)	GGAGGCCATGAAGGTTGACCAGCACG		Paired with 3' RACE primer
4	3' RACE primer (R)	Mixture of oligos: 5'-CTAATACGACTCACTATAGGGCAAGCAGTGGTAT CAACGCAGAGT-3' (0.4 μM) and 5'-CTAATACGACTCACTATAGGGC-3' (2 μM)	Same as Universal Primer A Mix (UPM) in SMARTer RACE cDNA Amplification Kit (Clontech)	

F, forward primer; R, reverse primer.

Supplementary Table 4. DNA probes used in EMSA experiments (Figure 4).

#	Name	Sequence 5'-3'	Note
1	Probe DNA B	CCGGGCGTTGCGCTCTCAAATTTCCACAGACACGACCCACGTCCAATTGTGAGTTTTCGCTGCGCCGTGAA TCGCACTAAAGAGCAGGAAAATAGTTAATTTCCAACAGTTAAATTGGAG	120 bp
2	Probe DNA B ΔDR1	CCGGGCGTTGCGCTCTCAAATTTCCACAGACACGACCCACGTCCAATTGTGAGTTTTCGCTGCGCCGTGAA TCATAGTTAATTTCCAACAGTTAAATTGGAG	102 bp

Direct repeat 1 (DR1) is highlighted in red.

Supplementary References

1. Goeke, S. *et al*. Alternative splicing of *lola* generates 19 transcription factors controlling axon guidance in *Drosophila*. *Nat. Neurosci.* **6**, 917-924 (2993).

2. Ohsako, T., Horiuchi, T., Matsuo, T., Komaya, S. & Aigaki, T. *Drosophila lola* encodes a family of BTB-transcription regulators with highly variable C-terminal domains containing zinc finger motifs. *Gene* **311**, 59-69 (2003).

3. Billeter, J.-C. *et al*. Isoform-specific control of male neuronal differentiation and behavior in *Drosophila* by the *fruitless* gene. *Curr. Biol.* **16**, 1063-1076 (2006).

4. Ito, H. *et al*. Fruitless recruits two antagonistic chromatin factors to establish single-neuron sexual dimorphism. *Cell* **149**, 1327-1338 (2012).
