# Supplementary material for: Partial proteasomal degradation of Lola triggers the male-to-female switch of a dimorphic courtship circuit
Source: Nat Commun. 2019 Jan 11;10:166. doi: 10.1038/s41467-018-08146-1 (PMC6329818; doi:10.1038/s41467-018-08146-1)
Supplement: Supplementary file 3 — Description of Additional Supplementary Files [file 41467_2018_8146_MOESM3_ESM.pdf]

**Description of Additional Supplementary Files**

File Name: Supplementary Movie 1
Description: A *robo1*$^{\Delta 4}$/*robo1*$^{\Delta 4}$ male courting a female, related to Supplementary Figure 11.
