# Supplementary material for: Partial proteasomal degradation of Lola triggers the male-to-female switch of a dimorphic courtship circuit
Source: Nat Commun. 2019 Jan 11;10:166. doi: 10.1038/s41467-018-08146-1 (PMC6329818; doi:10.1038/s41467-018-08146-1)
Supplement: Supplementary file 5 — Reporting Summary [file 41467_2018_8146_MOESM5_ESM.pdf]

nature research

Corresponding author(s): Kosei Sato, Daisuke Yamamoto

Reporting Summary

Nature Research wishes to improve the reproducibility of the work that we publish. This form provides structure for consistency and transparency in reporting. For further information on Nature Research policies, see Authors & Referees and the Editorial Policy Checklist.

Statistical parameters

When statistical analyses are reported, confirm that the following items are present in the relevant location (e.g. figure legend, table legend, main text, or Methods section).

n/a	Confirmed	
☐	☒	The exact sample size (*n*) for each experimental group/condition, given as a discrete number and unit of measurement
☐	☒	An indication of whether measurements were taken from distinct samples or whether the same sample was measured repeatedly
☐	☒	The statistical test(s) used AND whether they are one- or two-sided<br>*Only common tests should be described solely by name; describe more complex techniques in the Methods section.*
☒	☐	A description of all covariates tested
☐	☒	A description of any assumptions or corrections, such as tests of normality and adjustment for multiple comparisons
☐	☒	A full description of the statistics including central tendency (e.g. means) or other basic estimates (e.g. regression coefficient) AND variation (e.g. standard deviation) or associated estimates of uncertainty (e.g. confidence intervals)
☐	☒	For null hypothesis testing, the test statistic (e.g. *F*, *t*, *r*) with confidence intervals, effect sizes, degrees of freedom and *P* value noted<br>*Give P values as exact values whenever suitable.*
☒	☐	For Bayesian analysis, information on the choice of priors and Markov chain Monte Carlo settings
☒	☐	For hierarchical and complex designs, identification of the appropriate level for tests and full reporting of outcomes
☒	☐	Estimates of effect sizes (e.g. Cohen's *d*, Pearson's *r*), indicating how they were calculated
☐	☒	Clearly defined error bars<br>*State explicitly what error bars represent (e.g. SD, SE, CI)*

Our web collection on statistics for biologists may be useful.

Software and code

Policy information about availability of computer code

Data collection	No software was used for data collection.
Data analysis	Image J with relevant plug-in was used to analyze confocal microscopy images and to quantify protein bands from western blot films. VCode and VData softwares were used to analyze fly behavior. GENETYX-MAC was used to analyze DNA and protein sequence. Predicting DNA recognition (http://zf.princeton.edu/) was used for predicting DNA-binding sites for C2H2 Zinc Finger proteins (Lola29M/F). GraphPad Prism 7 was used for statistical analyses.

For manuscripts utilizing custom algorithms or software that are central to the research but not yet described in published literature, software must be made available to editors/reviewers upon request. We strongly encourage code deposition in a community repository (e.g. GitHub). See the Nature Research guidelines for submitting code & software for further information.

April 2018

Data

Policy information about availability of data

All manuscripts must include a data availability statement. This statement should provide the following information, where applicable:

- Accession codes, unique identifiers, or web links for publicly available datasets
- A list of figures that have associated raw data
- A description of any restrictions on data availability

The data that support the findings of this study are available from the corresponding author upon reasonable request.

Field-specific reporting

Please select the best fit for your research. If you are not sure, read the appropriate sections before making your selection.

☒ Life sciences ☐ Behavioural & social sciences ☐ Ecological, evolutionary & environmental sciences

For a reference copy of the document with all sections, see nature.com/authors/policies/ReportingSummary-flat.pdf

Life sciences study design

All studies must disclose on these points even when the disclosure is negative.

Sample size	No statistical methods were used to pre-determine sample sizes but our sample sizes are similar to those reported in previous publications. We clearly denoted the number of samples in the respective figures and/or figure legends.
Data exclusions	No data were excluded from our analyses.
Replication	At least 3 independent replicate experiments were performed. Statistical analyses were done to illustrate significance.
Randomization	No randomization was used.
Blinding	No blinding was done.

Reporting for specific materials, systems and methods

Materials & experimental systems

n/a	Involved in the study	
☒	☐	Unique biological materials
☐	☒	Antibodies
☐	☒	Eukaryotic cell lines
☒	☐	Palaeontology
☐	☒	Animals and other organisms
☒	☐	Human research participants

Methods

n/a	Involved in the study	
☒	☐	ChIP-seq
☒	☐	Flow cytometry
☒	☐	MRI-based neuroimaging

Antibodies

Antibodies used	Anti-LolaCOM, Lola-exon29 and FruMale antibodies were produced from immunized rabbit and guinea pig, respectively. Anti-alpha-Tubulin (Sigma, T6199), Anti-HA (Roche, 11867423001), Anti-V5 (Invitrogen, 46-0705), Anti-GFP (Sigma, A6455), nc82 (Hybridoma Bank), Anti-Deadpan (abcam, ab195173), Anti-Prospero (Hybridoma bank), Anti-Robo1 (Hybridoma Bank, 13C9), Anti-Cullin1 (Invitrogen, 71-8700), Anti-Ub-K48 (abcam, ab140601), mouse IgG (Invitrogen, 10400C) were purchased from manufacturers.
Validation	The specificity of the Anti-LolaCOM, Lola-exon29 and FruMale antibodies were validated by IHC and Western blotting, respectively.

Eukaryotic cell lines

Policy information about cell lines

Cell line source(s)	Drosophila S2 cells were purchased from Invitrogen (Catalog #R690-07).
Authentication	Authentication statement appears in the manufacturer's website.
Mycoplasma contamination	DAPI staining method did not detect any mycoplasma contamination.
Commonly misidentified lines (See ICLAC register)	Not applicable

Animals and other organisms

Policy information about studies involving animals; ARRIVE guidelines recommended for reporting animal research

Laboratory animals	Drosophila melanogaster. Sex and age of the examined animal are described in the respective figure or figure legend.
Wild animals	Not applicable
Field-collected samples	Not applicable
